# Using Natural Language Processing and Machine Learning to Preoperatively Predict Lymph Node Metastasis for Non–Small Cell Lung Cancer With Electronic Medical Records: Development and Validation Study

**DOI:** 10.2196/35475

**Published:** 2022-04-25

**Authors:** Danqing Hu, Shaolei Li, Huanyao Zhang, Nan Wu, Xudong Lu

**Affiliations:** 1 College of Biomedical Engineering and Instrumental Science Zhejiang University Hangzhou China; 2 Department of Thoracic Surgery II Peking University Cancer Hospital and Institute Beijing China

**Keywords:** non–small cell lung cancer, lymph node metastasis prediction, natural language processing, electronic medical records, lung cancer, prediction models, decision making, machine learning, algorithm, forest modeling

## Abstract

**Background:**

Lymph node metastasis (LNM) is critical for treatment decision making of patients with resectable non–small cell lung cancer, but it is difficult to precisely diagnose preoperatively. Electronic medical records (EMRs) contain a large volume of valuable information about LNM, but some key information is recorded in free text, which hinders its secondary use.

**Objective:**

This study aims to develop LNM prediction models based on EMRs using natural language processing (NLP) and machine learning algorithms.

**Methods:**

We developed a multiturn question answering NLP model to extract features about the primary tumor and lymph nodes from computed tomography (CT) reports. We then combined these features with other structured clinical characteristics to develop LNM prediction models using machine learning algorithms. We conducted extensive experiments to explore the effectiveness of the predictive models and compared them with size criteria based on CT image findings (the maximum short axis diameter of lymph node >10 mm was regarded as a metastatic node) and clinician’s evaluation. Since the NLP model may extract features with mistakes, we also calculated the concordance correlation between the predicted probabilities of models using NLP-extracted features and gold standard features to explore the influence of NLP-driven automatic extraction.

**Results:**

Experimental results show that the random forest models achieved the best performances with 0.792 area under the receiver operating characteristic curve (AUC) value and 0.456 average precision (AP) value for pN2 LNM prediction and 0.768 AUC value and 0.524 AP value for pN1&N2 LNM prediction. And all machine learning models outperformed the size criteria and clinician’s evaluation. The concordance correlation between the random forest models using NLP-extracted features and gold standard features is 0.950 and improved to 0.984 when the top 5 important NLP-extracted features were replaced with gold standard features.

**Conclusions:**

The LNM models developed can achieve competitive performance using only limited EMR data such as CT reports and tumor markers in comparison with the clinician’s evaluation. The multiturn question answering NLP model can extract features effectively to support the development of LNM prediction models, which may facilitate the clinical application of predictive models.

## Introduction

Lung cancer remains the leading cause of cancer death worldwide, representing approximately 1 in 5 (18.0%) cancer deaths [1]. Non–small cell lung cancer (NSCLC) accounts for about 84% of lung cancer cases, and its 5-year relative survival rate is only 25.0% [2], making it one of the biggest threats to human health.

Staging of NSCLC is a process to determine the extent of the cancer and is critical to prognosis evaluation and treatment decision making [3,4]. The TNM stage classification [5] is the most widely used staging method in clinical practice; it describes the anatomic extent of a tumor from 3 aspects (ie, T for extent of the primary tumor, N for involvement of lymph nodes, M for distant metastases). For patients with resectable NSCLC, preoperative confirmed N2 (a type of N stage) lymph node metastasis (LNM) indicates neoadjuvant therapy should be given before surgery to achieve the best clinical practice [3]. Currently, various advanced noninvasive diagnostic modalities are available for N staging like chest computed tomography (CT) and positron emission tomography–computed tomography (PET-CT). In clinical practice, clinicians commonly use a size criterion (ie, the maximum short axis diameter of lymph node >10 mm on CT scan) to discriminate LNM from benign nodes and yield 55% sensitivity [6]. Another criterion is the maximum standardized uptake value (SUVmax) of lymph node >2.5 on PET-CT scan, which has an 81% sensitivity [7]. Invasive methods such as mediastinoscopy and endobronchial ultrasound-guided transbronchial needle aspiration have better diagnostic abilities than noninvasive methods. However, these methods are mainly for lymph nodes with indications and not suitable for patients with severe comorbidities, so they are not routinely used in clinical practice [8]. One study analyzed data from 9 clinical trials and found nearly 38% of patients were misclassified in comparison with their pathological N staging [9]. Therefore, new reliable LNM prediction methods are required to alleviate this clinical dilemma.

For precise staging, researchers explored using statistical analysis or machine learning methods to learn nontrivial knowledge between the comprehensive patient features and LNM status [8,10-16]. Recently, with the rapid development of hospital information systems, a large volume of electronic medical records (EMR) has become available, and it contains almost all clinical features about patients. However, some important features are recorded in the narratives in free text, such as the size of the tumor and lymph node, tumor density, pleural indentation, etc, which hinders their direct use. Manual extraction is time-consuming and error-prone. So, one big challenge is how to extract this information effectively to support subsequent tasks like LNM prediction [17]. A review by Garg et al [18] found studies in which users were automatically prompted to use the system achieved better performance in comparison with those in which users were required to actively initiate the system. The finding implicitly indicates that the duplicative data entry activity may explain why the predictive models are not widely adopted in the clinic despite their potential to improve diagnostic accuracy. Furthermore, with the prevalence of machine learning models, more features are required for analysis, making the clinical application of the models more difficult [19-21].

Natural language processing (NLP) offers the opportunity to automatically extract information to support the application of predictive models [17,22]. Many studies used rule-based, machine learning, or deep learning methods to extract the cancer-related information from free-text EMR data [22-29], but only a few included further elaboration on how to exploit the extracted information. Chen et al [30] extracted information from various clinical notes including CT reports and operative notes to calculate the Cancer of the Liver Italian Program score. Martinez et al [31] extracted information from pathology reports to calculate the TNM and Australian clinicopathological stage of colorectal cancer. Castro et al [32] developed an NLP system for automated breast imaging reporting and data system (BI-RADS) categories extraction from breast radiology reports. Bozkurt et al [33,34] developed an information extraction pipeline to extract information from mammography reports to predict the malignancy of breast cancer. Sui et al [35] constructed an NLP-based feature generalizing to extract features from free-text EMR data and provided the stage of lung cancer using a Bayesian reasoning network. Yuan et al [36] used NLP tools to extract multiple features from EMRs to estimate survival for patients with lung cancer. Although many studies have explored how to extract the cancer-related information from various types of free-text narratives and some also exploit the extracted information for cancer risk evaluation, diagnosis, and pathological staging, few studies exploit the extracted information from radiological reports for preoperative LNM prediction, especially for NSCLC.

In this study, we aim to use EMR data to develop LNM prediction models for NSCLC patients. We first developed a multiturn question answering NLP model to extract the features from CT reports and then combined these features with other clinical characteristics to develop the predictive models. Since the NLP model may produce imperfect extraction results, we also conducted experiments to compare the predicted probabilities between models using NLP-extracted features and gold standard features.

## Methods

### Patients

We retrospectively analyzed EMR data of 794 patients who underwent surgical resection for NSCLC with systematic mediastinal lymphadenectomy at the Department of Thoracic Surgery II of Peking University Cancer Hospital from 2010 to 2018. All patients underwent contrast-enhanced chest CT images within 2 months before surgical resection. We excluded the patients with preoperative chemotherapy or radiotherapy. The collected EMR includes demographic information, medical history, CT reports, preoperative serum tumor markers, and pathology reports, which can be analyzed to develop the prediction model. For each patient, we also collected the clinical staging that clinicians evaluated before surgery as the baseline to compare with the LNM prediction models.

### Ethics Approval

This study was approved by the Ethics Committee of Peking University Cancer Hospital (2019KT59).

### Clinical and Pathological LNM Evaluation

In this study, all included patients underwent systematic mediastinal lymphadenectomy during surgical resection. The lymph node tissues were examined by pathologists, and the metastasis results were recorded in the postoperative pathology reports. We reviewed the pathology reports to determine the LNM status and label the pathological N (pN) stage (pN0/pN1/pN2) for each patient based on the 8th edition TNM stage classification [5] as the gold standard. We also used the size criterion (ie, the maximum short axis diameter of lymph node >10 mm on CT scan as positive) to label the clinical N (cN) stages (cN0/cN1/cN2) based on the CT-reported lymph node size. Moreover, we collected the cN stages, which were determined preoperatively by a thoracic surgeon using all available patient data including the information used in this study. The thoracic surgeon has 10 years of experience in lung cancer surgery. The cN stages determined by the size criterion and the thoracic surgeon were regarded as the baselines.

### NLP Feature Extraction

As one of the most important preoperative examinations, CT reports record valuable information about the tumors and lymph nodes, which is of paramount importance for staging. However, the free-text nature of CT reports makes it difficult to understand and analyze them using computer programs. In our previous work [27], we developed an information extraction system composed of named entity recognition, relation classification, and postprocessing modules to extract valuable information in a pipeline manner. However, in this pipeline, the subsequent tasks would be influenced by the outputs of former tasks, which may affect the performance of the whole system. Therefore, to alleviate this problem, we applied a multiturn question answering (MTQA) [37] approach to extract information from CT reports in this study. Using the MTQA strategy, we can encode the relation into the question query and jointly model entity and relation in a natural question answering way.

**Figure 1:**
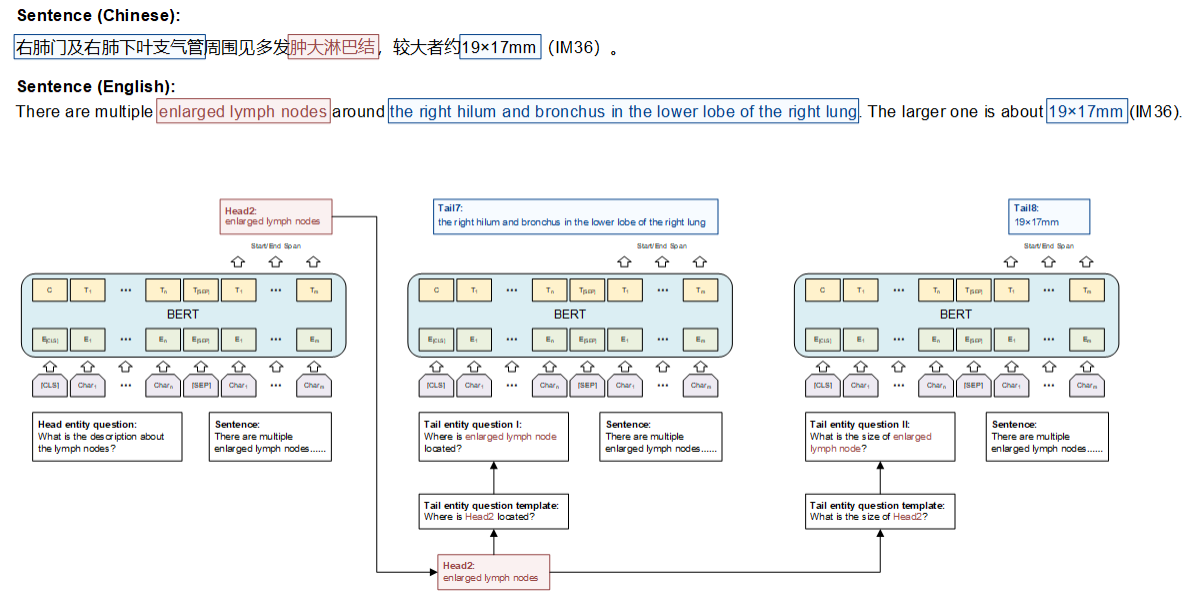
A case of multiturn question answering application. BERT: bidirectional encoder representations from transformers.

Specifically, we first defined 10 questions related to the primary tumor and lymph nodes. All questions are listed in Table 1. Note that there are 2 types of questions (ie, head entity questions and tail entity question templates). In the model training stage, we inserted the annotated head entities into the slots in the tail entity question templates as the tail entity questions. We then used 2 special tokens (ie, CLS and SEP) to concatenate the questions and sentences in the reports as the inputs and annotated entities as the answers to conduct the bidirectional encoder representations from transformers (BERT) model training. In the model test stage, we first concatenated the head entity questions and sentences in the reports as the inputs and applied the trained MTQA model to extract the head entities (ie, tumor and lymph node). If there were any head entities recognized, we inserted the extracted head entities into the slots in the tail entity question templates as the tail entity questions and combined them with sentences in the reports as the inputs to drive the tail entity extraction. A case of the MTQA application is shown in Figure 1. Finally, the extracted head and tail entities are organized as triples, and a rule-based postprocessing algorithm proposed in the previous work [27] is used to process the triples to obtain the standardized NLP-extracted features. Furthermore, the NLP-extracted features were manually reviewed and corrected by a clinician based on the report contents as the gold standard features. In this study, we used BERT [38], an advanced pretrained language representation model, to tag the answer for each question.

**Table 1:** Questions and entity types for natural language processing–extracted features.

Question (Chinese)			Question (English)		Answer notation		Entity type
**Head entity question**							
	原发肿物的相关描述是什么？	What is the description about the primary tumor?		Head1		Tumor	
	淋巴结的相关描述是什么？	What is the description about the lymph nodes?		Head2		Lymph node	
**Tail entity question template**							
	Head1 位于什么地方？	Where is Head1 located?		Tail1		Location	
	Head1 的大小是多少？	What is the size of Head1?		Tail2		Size	
	Head1 的形状是什么？	What is the shape of Head1?		Tail3		Shape	
	Head1 的密度是什么？	What is the density of Head1?		Tail4		Density	
	与Head1 相关的胸膜侵犯的描述是什么？	What is the description about the pleura invasion related to Head1?		Tail5		Pleura	
	与Head1 相关的血管侵犯的描述是什么？	What is the description about the vessel invasion related to Head1?		Tail6		Vessel	
	Head2 位于什么地方？	Where is Head2 located?		Tail7		Location	
	Head2 的大小是多少？	What is size of Head2?		Tail8		Size	

### LNM Prediction

Six machine learning algorithms were applied to develop the LNM prediction models, including logistic regression (LR) [39], L2-logistic regression (L2-LR) [40], random forest (RF) [41], LightGBM (LGBM) [42], support vector machine (SVM) [43], and artificial neural network (ANN) [44]. LR is the conventional classification method, and L2-LR is the LR with the L2 regularization for parameters. RF and LGBM are ensemble methods but with different ways to combine the weak decision trees. SVM is a classical algorithm that constructs hyperplanes in a high- or infinite-dimensional space to classify samples. ANN is a supervised learning algorithm that can learn nonlinear functions between features and targets. LR and L2-LR have good interpretability because the predicted results can be calculated by a simple linear function and a sigmoid transformation. RF and LGBM are also interpretable, in which they can provide the feature importance.

### Experimental Setup

In this study, we used the Whole Word Masking version of BERT [45] pretrained on the Chinese Wikipedia corpus as the tagging model in the MTQA. An additional 359 annotated CT reports from our previous work were used to develop and evaluate the MTQA model. We randomly split 70% of CT reports as the training set, 10% as the validation set, and 20% as the test set. A total of 100 of these reports were each annotated by 2 biomedical informatics engineers to calculate the interannotator agreement score using the kappa score. Pipeline methods with bidirectional long short-term memory (BiLSTM) and BERT were selected as the baseline. To obtain the NLP-extracted features for LNM prediction, the MTQA model developed on the 359 reports was used to process the 794 CT reports of included patients. Subsequently, the NLP-extracted features were manually reviewed and corrected by a clinician as the gold standard features.

Univariate analysis was performed using the Mann-Whitney *U* test for continuous features and Pearson chi-square test for categorical features. *P*<.05 was considered statistically significant. To obtain robust experimental results, a 10-fold cross-validation strategy was first performed on the total data set. The 10-fold cross-validation randomly split the data set into 10 subsets. Each subset was considered as the independent test set and the remaining 9 subsets were considered as the training set. During each fold, a 5-fold cross-validation was applied on the training set to find the optimal hyperparameters for the machine learning algorithms by a grid search. When the optimal hyperparameters were selected, we retrained the prediction model on the training set and tested it on the test set to obtain the final predictive performance. Using this strategy, we can ensure that the test set is always invisible during the model training and hyperparameter tuning and obtain the predicted probability for each case. The hyperparameter spaces are as follows:

- LR: tol ∈ {1e–3, 1e–4, 1e–5}, max_iter ∈ {500, 1000}
- L2-LR: C ∈ {10, 1, 0.1}, tol ∈ {1e–3, 1e–4, 1e–5}, max_iter ∈ {500, 1000}
- RF: n_estimators ∈ {50, 100, 200}, max_depth ∈ {2, 3}, min_samples_leaf ∈ {1, 2}
- LGBM: n_estimators ∈ {50, 100, 200}, max_depth ∈ {2, 3}, num_leaves ∈ {20, 31, 50}, min_child_samples ∈ {1, 2, 3}, reg_alpha ∈ {2, 3}
- SVM: C ∈ {10, 1, 0.1, 0.01}, kernel ∈ {‘linear,’ ‘rbf,’ ‘poly’}, tol ∈ {1e–3, 1e–4, 1e–5}
- ANN: hidden_layer_sizes ∈ {5, 10, 30}, learning_rate ∈ {1e–2, 1e–3, 1e–4}, alpha ∈ {1e–3, 1e–4, 1e–5}

We applied the receiver operating characteristic (ROC) curve to evaluate the diagnostic performances of the machine learning models. Besides the ROC curve, we also used the precision-recall (PR) curve to test the models because the ROC curve pays attention to sensitivity and specificity but ignores precision. The mean area under the receiver operating characteristic curve (AUC) and average precision (AP) values with standard derivations were calculated based on the 10-fold cross-validation results. We also drew the ROC curves and PR curves to compare with the size criterion (maximum short axis diameter of lymph node >10 mm on CT) and the clinician’s evaluation. All LNM prediction models were developed using the Scikit-learn 0.24.1 and LightGBM 3.2.0 Python packages. All statistical analyses were conducted using SciPy 1.6.2 Python package.

## Results

### Patient Characteristics

Table 2 shows the characteristics of all 794 patients. Univariate analysis was performed for all collected features, and 13.2% (105/794) of patients had pN2 LNM. Sex, age, drinking history, family history, and disease history are not significantly associated with the pN2. The pN2 occurred more frequently in smokers (*P*=.04). The long and short axis diameters of the tumor in pN2 patients are significantly larger than those in pN0 and pN1 patients (both *P*<.001). Patients with solid nodules are more likely to have pN2 (*P*<.001). Other morphological characteristics of tumor-like lobulation and pleural indentation are more likely to occur in pN2 patients (*P*=.006 and *P*=.003, respectively), but spiculation and vessel invasion present no significant differences between pN2 and other patients. Using 10 mm as the size criterion, the maximum long and short axis diameters of the hilar and mediastinal lymph nodes show significant differences between the 2 groups (*P*=.008, *P*<.001, *P*<.001, and *P*<.001, respectively). Among all 6 serum tumor biomarkers, carcinoembryonic antigen (CEA), carbohydrate antigen 12-5 (CA125), and neuron-specific enolase (NSE) show significant differences between the 2 groups (*P*<.001, *P*<.001, and *P*=.048, respectively).

**Table 2:** Patient characteristics.

		Total (n=794)	LNM^a^ status			*P* value
			pN2^b^ (n=105)	pN0^c^ or pN1^d^ (n=689)		
Age (years), mean (SD)		60.92 (51.48 to 70.36)	60.87 (51.87 to 69.86)	60.93 (51.42 to 70.44)	.45	
**Sex, n (%)**		—^e^	—	—	.06	
	Male	397	62	335	—	
	Female	397	43	354	—	
**Smoking history, n (%)**		—	—	—	.04	
	Yes	337	55	282	—	
	No	457	50	407	—	
**Drinking history, n (%)**		—	—	—	.94	
	Yes	183	25	158	—	
	No	611	80	531	—	
**Family history, n (%)**		—	—	—	.32	
	Yes	137	14	123	—	
	No	657	91	566	—	
**Hypertension, n (%)**		—	—	—	.18	
	Yes	232	37	195	—	
	No	562	68	494	—	
**Diabetes, n (%)**		—	—	—	.25	
	Yes	84	15	69	—	
	No	710	90	620	—	
**Pulmonary tuberculosis, n (%)**		—	—	—	.33	
	Yes	33	2	31	—	
	No	761	103	658	—	
**Cardiovascular disease, n (%)**		—	—	—	.06	
	Yes	36	9	27	—	
	No	758	96	662	—	
**Cerebrovascular disease, n (%)**		—	—	—	.35	
	Yes	29	6	23	—	
	No	765	99	666	—	
**Tumor location^f^, n (%)**		—	—	—	.22	
	RUL^g^	249	27	222	—	
	RML^h^	59	4	55	—	
	RLL^i^	150	18	132	—	
	LUL^j^	185	31	154	—	
	LLL^k^	126	21	105	—	
	Other	25	4	21	—	
TLA^f,l^, median (IQR)		2.61 (1.20 to 4.01)	3.02 (1.64 to 4.39)	2.55 (1.15 to 3.94)	<.001	
TSA^f,m^, median (IQR)		2.03 (0.88 to 3.18)	2.38 (1.27 to 3.48)	1.98 (0.83 to 3.13)	<.001	
**Spiculation^f^, n (%)**		—	—	—	.08	
	Yes	255	42	213	—	
	No	539	63	476	—	
**Lobulation^f^, n (%)**		—	—	—	<.001	
	Yes	211	48	163	—	
	No	583	57	526	—	
**Tumor density^f^, n (%)**		—	—	—	<.001	
	pGGO^n^	124	0	124	—	
	mGGO^o^	96	3	93	—	
	Solid nodule	574	102	472	—	
**Vessel invasion^f^, n (%)**		—	—	—	.87	
	Yes	52	6	46	—	
	No	742	99	643	—	
**Pleural indentation^f^, n (%)**		—	—	—	.001	
	Yes	406	70	336	—	
	No	388	35	353	—	
**HLNLA^f,p^, n (%)**		—	—	—	.008	
	>10 mm	148	30	118	—	
	≤10 mm	646	75	571	—	
**HLNSA^f,q^, n (%)**		—	—	—	<.001	
	>10 mm	66	19	47	—	
	≤10 mm	728	86	642	—	
**MLNLA^f,r^, n (%)**		—	—	—	<.001	
	>10 mm	191	50	141	—	
	≤10 mm	603	55	548	—	
**MLNSA^f,s^, n (%)**		—	—	—	<.001	
	>10 mm	72	27	45	—	
	≤10 mm	722	78	644	—	
CEA^t^, median (IQR)		5.31 (–6.66 to 17.27)	12.66 (–8.44 to 33.76)	4.18 (–5.17 to 13.54)	<.001	
CA199^u^, median (IQR)		14.41 (–3.24 to 32.06)	15.80 (–5.08 to 36.68)	14.20 (–2.90 to 31.29)	.47	
CA125^v^, median (IQR)		14.46 (0.03 to 28.90)	19.88 (–5.56 to 45.32)	13.64 (1.96 to 25.32)	<.001	
NSE^w^, median (IQR)		15.81 (8.85 to 22.78)	16.26 (10.19 to 22.33)	15.75 (8.66 to 22.83)	.048	
Cyfra211^x^, median (IQR)		3.20 (–0.23 to 6.62)	3.55 (–0.64 to 7.75)	3.14 (–0.15 to 6.43)	.06	
SCCAg^y^, median (IQR)		0.96 (–0.16 to 2.08)	1.18 (–0.62 to 2.99)	0.93 (–0.04 to 1.90)	.14	

^a^LNM: lymph node metastasis.

^b^pN2: pathological N stage 2.

^c^pN0: pathological N stage 0.

^d^pN1: pathological N stage 1.

^e^Not applicable.

^f^Features recorded in computed tomography reports.

^g^RUL: right upper lobe.

^h^RML: right middle lobe.

^i^RLL: right lower lobe.

^j^LUL: left upper lobe.

^k^LLL: left lower lobe.

^l^TLA: tumor long axis.

^m^TSA: tumor short axis

^n^pGGO: pure ground glass opacity.

^o^mGGO: mixed ground glass opacity.

^p^HLNLA: hilar lymph node long axis.

^q^HLNSA: hilar lymph node short axis.

^r^MLNLA: mediastinal lymph node long axis.

^s^MLNSA: mediastinal lymph node short axis.

^t^CEA: carcinoembryonic antigen.

^u^CA199: carbohydrate antigen 19-9.

^v^CA125: carbohydrate antigen 12-5.

^w^NSA: neuron-specific enolase.

^x^Cyfra211: cytokeratin 19-fragments.

^y^SCCAg: squamous cell carcinoma antigen.

### Performance of pN2 LNM Prediction Models

**Figure 2:**
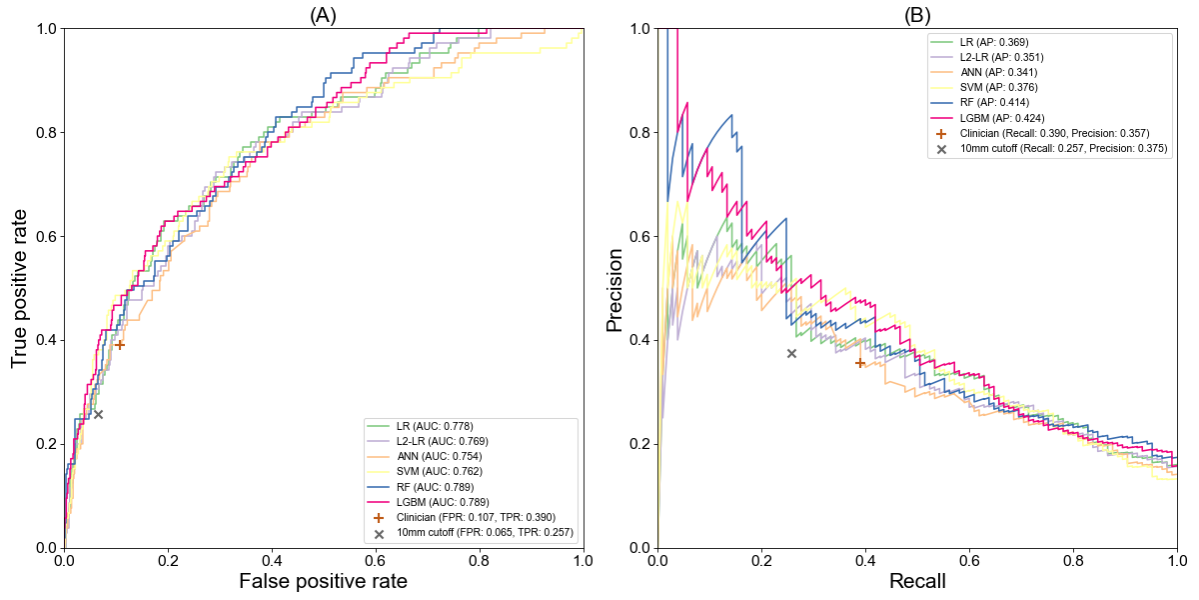
The receiver operating characteristic curve (A) and precision-recall curves (B) of pN2 prediction models.

As preoperative confirmed N2 indicating neoadjuvant therapy should be given before surgery, we first developed machine learning models to predict the pN2 LNM. We regarded the pN2 patients as positive and pN0 and pN1 patients as negative to train the predictive models. To obtain reliable models, we used the gold standard features instead of NLP-extracted features in this section. Table 3 shows the performances of all models. The RF model achieved the highest averaged AUC value with 0.792 and the LGBM model achieved the highest averaged AP value with 0.457 while all models’ 95% CI are overlapping with each other. The LR obtained a competitive performance in comparison with ANN and SVM. The L2-LR did not obtain improvements in AUC value and AP value compared with the LR. To compare with the size criterion and clinician’s evaluation, we used the probabilities predicted during the 10-fold cross-validation to draw the ROC and PR curves. Figure 2 shows the ROC curves and PR curves of pN2 prediction models and the results of the size criterion and clinician’s evaluation. From Figure 2 we can notice all the ROC curves and PR curves are above the points of size criterion and clinician’s evaluation, which indicates the developed pN2 prediction models not only have better discriminative ability than the diagnostic size criterion used in the clinical practice but also may exceed the clinician in pN2 LNM evaluation.

**Table 3:** Performances of pN2 lymph node metastasis prediction models.

Model	AUC^a^			AP^b^		
	Mean	SD	95% CI	Mean	SD	95% CI
LR^c^	0.778	0.041	0.747-0.809	0.442	0.075	0.385-0.499
L2-LR^d^	0.768	0.038	0.739-0.796	0.413	0.072	0.359-0.467
ANN^e^	0.769	0.051	0.730-0.808	0.434	0.095	0.363-0.506
SVM^f^	0.771	0.071	0.718-0.825	0.453	0.084	0.389-0.516
RF^g^	0.792	0.042	0.760-0.825	0.456	0.075	0.399-0.512
LGBM^h^	0.787	0.044	0.755-0.820	0.457	0.101	0.381-0.534

^a^AUC: area under the receiver operating characteristic curve.

^b^AP: average precision.

^c^LR: logistic regression.

^d^L2-LR: L2-logistic regression.

^e^ANN: artificial neural network.

^f^SVM: support vector machine.

^g^RF: random forest.

^h^LGBM: LightGBM.

### Performance of pN1&N2 LNM Prediction Models

**Figure 3:**
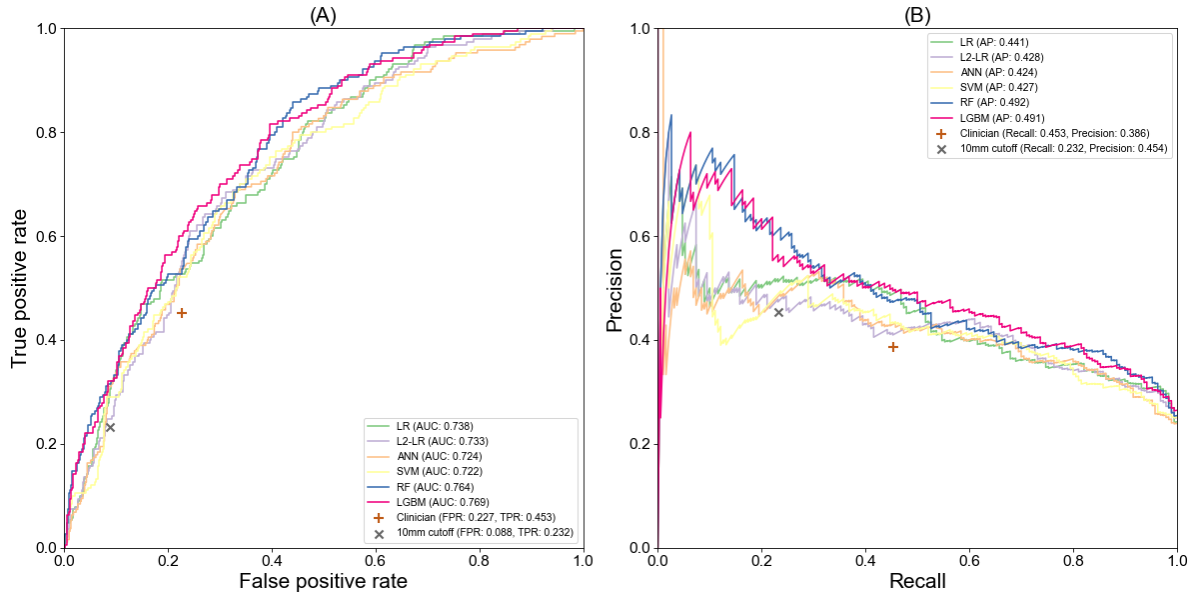
The receiver operating characteristic curve (A) and precision-recall curves (B) of pN1&N2 prediction models.

Besides predicting pN2 LNM, we also developed machine learning models to predict the pN1&N2 LNM by regarding patients with pN1 or pN2 LNM as positive. The model training and evaluation processes are the same as pN2 LNM prediction. Table 4 shows the performances of the machine learning models for pN1&N2 LNM prediction. LGBM obtained the highest averaged AUC value with 0.771. The RF model achieved a comparable performance in comparison with LGBM. As in pN2 prediction, LGBM and RF obtained better predictive performances than other models. Figure 3 shows the ROC curves and PR curves of pN1&N2 LNM prediction models. The curves of the machine learning models are also all above the points of the size criterion and clinician’s evaluation.

**Table 4:** Performances of pN1&N2 lymph node metastasis prediction models.

Model	AUC^a^			AP^b^		
	Mean	SD	95% CI	Mean	SD	95% CI
LR^c^	0.740	0.035	0.714-0.766	0.467	0.058	0.423-0.510
L2-LR^d^	0.736	0.044	0.704-0.769	0.465	0.058	0.422-0.509
ANN^e^	0.734	0.047	0.698-0.770	0.479	0.087	0.413-0.545
SVM^f^	0.735	0.023	0.717-0.752	0.474	0.047	0.439-0.509
LGBM^g^	0.768	0.030	0.745-0.791	0.524	0.044	0.491-0.557
RF^h^	0.771	0.026	0.752-0.791	0.524	0.057	0.481-0.567

^a^AUC: area under the receiver operating characteristic curve.

^b^AP: average precision.

^c^LR: logistic regression.

^d^L2-LR: L2-logistic regression.

^e^ANN: artificial neural network.

^f^SVM: support vector machine.

^g^RF: random forest.

^h^LGBM: LightGBM.

### Feature Importance

Among all machine learning models, the LR, L2-LR, RF, and LGBM can provide the feature importance. Table 5 shows the top 10 important features of LR, L2-LR, RF, and LGBM for pN2 LNM prediction. The features were ranked by averaging the weights of models developed from 10-fold cross validation. Note that the LR and L2-LR models provide weights with signs, so we used the absolute values to rank the features. Because the weight magnitudes from different models vary greatly, we used the averaged rankings of features, but not the averaged weights, to find the most important features among the 4 types of models. The CEA is ranked as the most important feature to increase the risk of pN2 LNM by all models. Features recorded in CT reports account for at least half of the top 10 important features, indicating these features are of great importance for pN2 LNM prediction.

**Table 5:** Top 10 important features for pN2 lymph node metastasis prediction.

Rank	LR^a^			L2-LR^b^			RF^c^			LGBM^d^			All
	Feature	Weight	Feature		Weight	Feature		Weight	Feature		Weight		
1	pGGO^e,f^	–10.383	CEA^g^		3.530	CEA		0.229	CEA		46.0	CEA	
2	CEA	6.010	CA125^h^		3.067	CA125		0.094	Age		23.3	Solid nodule^f^	
3	CA125	4.728	pGGO^f^		–1.799	Solid nodule^f^		0.094	Solid nodule^f^		18.8	CA125	
4	Solid nodule^f^	3.683	Solid nodule^f^		1.773	MLNSA^f,i^		0.073	TLA^f,j^		17.6	Age	
5	TLA^f^	–2.701	Age		–1.315	MLNLA^f,k^		0.072	TSA^f,l^		15.1	MLNLA^f^	
6	Age	–1.908	SCCAg^m^		0.944	TLA^f^		0.054	CA125		13.3	TLA^f^	
7	SCCAg	1.763	MLNLA^f^		0.896	TSA^f^		0.048	Cyfra211^n^		12.9	pGGO^f^	
8	mGGO^f,o^	1.759	Pleural indentation^f^		0.836	Cyfra211		0.038	NSE^p^		12.7	SCCAg	
9	RML^f,q^	–1.729	Cardiovascular disease		0.807	SCCAg		0.037	MLNLA^f^		11.6	Lobulation^f^	
10	TSA^f^	1.601	Lobulation^f^		0.725	Lobulation^f^		0.036	SCCAg		9.0	TSA^f^	

^a^LR: logistic regression.

^b^L2-LR: L2-logistic regression.

^c^RF: random forest.

^d^LGBM: LightGBM.

^e^pGGO: pure ground glass opacity.

^f^Features recorded in computed tomography reports.

^g^CEA: carcinoembryonic antigen.

^h^CA125: carbohydrate antigen 12-5.

^i^MLNSA: mediastinal lymph node short axis.

^j^TLA: tumor long axis.

^k^MLNLA: mediastinal lymph node long axis.

^l^TSA: tumor short axis.

^m^SCCAg: squamous cell carcinoma antigen.

^n^Cyfra211: cytokeratin 19-fragments.

^o^mGGO: mixed ground glass opacity.

^p^NSE: neuron-specific enolase.

^q^RML: right middle lobe.

### NLP-Extracted Features Versus Gold Standard Features

In this study, we applied the MTQA model to extract important features from CT reports to support the development of LNM prediction models. In this section, we first conduct experiments to explore the effectiveness of the MTQA model on feature extraction and then analyze the influence of imperfect extraction results on LNM prediction.

We used an additional 359 annotated CT reports to develop the MTQA model. The interannotator agreement score was 0.937 based on the 100 reports annotated by 2 annotators. Table 6 shows the performances of the MTQA model and the pipeline models on the test set. We can notice that the BERT-MTQA model achieved significant improvement compared with the pipeline models.

Table 7 illustrates the performance of the BERT-MTQA model on the 794 CT reports of included patients. We can notice that the accuracy values of all extracted features are higher than 0.90. The F1 scores are higher than 0.90 except for lobulation, tumor density, vessel invasion, and hilar lymph node long axis. For the NLP-extracted features ranked in the top 10 important features, the mediastinal lymph node long axis (MLNLA), tumor long axis (TLA), and tumor short axis (TSA) obtained good accuracy values and F1 scores, but the F1 scores of tumor density and lobulation are not higher than 0.90.

**Figure 4:**
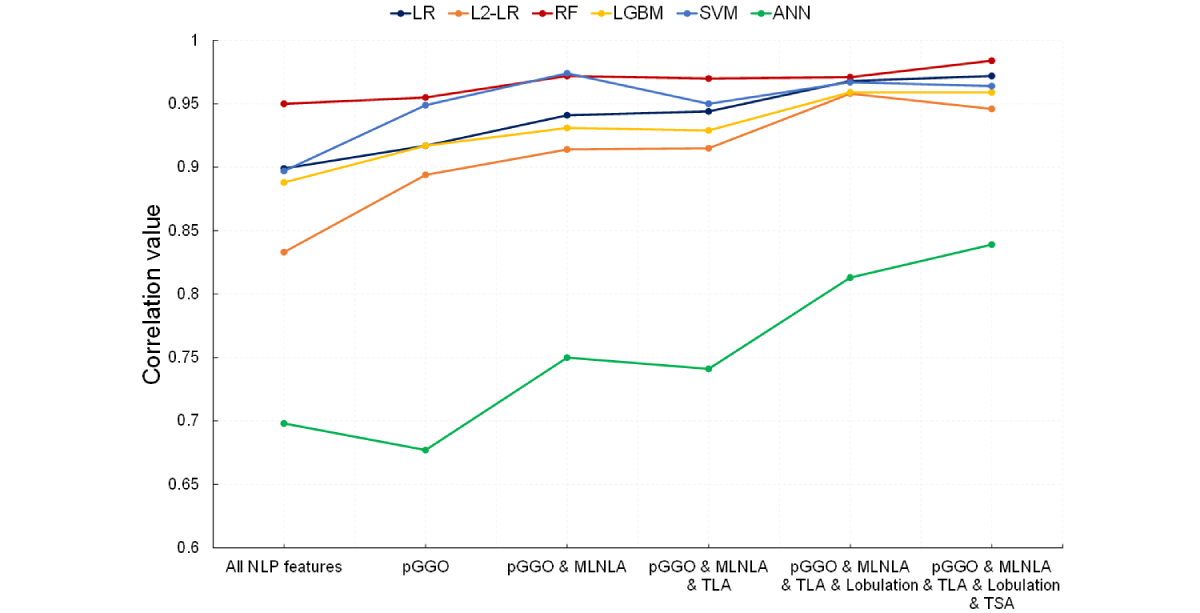
Concordance correlation values between pN2 prediction models using complete and partial gold standard features. LR: logistic regression; L2-LR: L2-logistic regression; RF: random forest; LGBM: LightGBM; SVM: support vector machine; ANN: artificial neural network: NLP: natural language processing; pGGO: pure ground glass opacity; MLNLA: mediastinal lymph node long axis; TLA: tumor long axis; TSA: tumor short axis.

In this study, the MTQA model generates imperfect extractions, which may influence the subsequent application. To analyze the influence on the pN2 LNM prediction, we calculated the Pearson correlation between the predicted probabilities of models using NLP-extracted features and gold standard features. Moreover, we also replaced the NLP-extracted feature with the gold standard feature one by one according to their importance in Table 5 to explore the changes in the consistency. Figure 4 shows the concordance correlations of the pN2 LNM prediction models. The RF model obtained a high concordance correlation with 0.950 when using all NLP-extracted features in comparison with using gold standard features, and the correlation increased to 0.984 when replacing top 5 important NLP-extracted features. The correlation values of the LR, L2-LR, LGBM, and SVM models were more influenced by using the NLP-extracted features. With the replacement of gold standard features, the correlation values gradually increased and exceeded 0.950. The ANN model did not achieve a good concordance correlation even when the top 5 important NLP-extracted features were replaced.

**Table 6:** Performance of the multiturn question answering model and baseline models.

Feature	BiLSTM^a^-pipeline				BERT^b^-pipeline				BERT-MTQA^c^		
	P^d^	R^e^	F^f^	P		R	F	P		R	F
Tumor density	0.882	0.625	0.732	0.889		0.667	0.762	0.938		0.938	0.938
MLNLA^g^	1.000	0.640	0.780	1.000		0.720	0.837	1.000		0.960	0.980
TLA^h^	0.967	0.892	0.928	0.984		0.938	0.961	0.984		0.954	0.969
Lobulation	0.889	0.533	0.667	0.909		0.667	0.769	1.000		0.867	0.929
TSA^i^	0.967	0.892	0.928	0.984		0.938	0.961	0.984		0.954	0.969
MLNSA^j^	1.000	0.750	0.857	1.000		0.750	0.857	1.000		0.938	0.968
Pleural indentation	0.931	0.818	0.871	0.964		0.818	0.885	1.000		0.848	0.918
Tumor location	0.984	0.897	0.938	0.968		0.897	0.931	0.985		0.985	0.985
Spiculation	1.000	0.727	0.842	1.000		0.773	0.872	1.000		1.000	1.000
Vessel invasion	1.000	0.111	0.200	1.000		0.222	0.364	1.000		0.556	0.714
HLNLA^k^	1.000	0.778	0.875	1.000		0.833	0.909	1.000		1.000	1.000
HLNSA^l^	1.000	0.750	0.857	1.000		0.750	0.857	1.000		1.000	1.000
Average	0.968	0.701	0.790	0.975		0.748	0.830	0.991		0.917	0.948

^a^BiLSTM: bidirectional long short-term memory.

^b^BERT: bidirectional encoder representations from transformers.

^c^MTQA: multiturn question answering.

^d^P: precision.

^e^R: recall.

^f^F: F1 score.

^g^MLNLA: mediastinal lymph node long axis.

^h^TLA: tumor long axis.

^i^TSA: tumor short axis.

^j^MLNSA: mediastinal lymph node short axis.

^k^HLNLA: hilar lymph node long axis.

^l^HLNSA: hilar lymph node short axis.

**Table 7:** Performance of the multiturn question answering model for feature extraction.

Feature	Accuracy	Precision	Recall	F1 score
Tumor density	0.940	0.875	0.915	0.893
MLNLA^a^	0.965	0.927	0.927	0.927
TLA^b^	0.974	0.974	0.974	0.974
Lobulation	0.923	0.993	0.716	0.832
TSA^c^	0.972	0.972	0.972	0.972
MLNSA^d^	0.986	0.918	0.931	0.924
Pleural indentation	0.917	0.903	0.938	0.920
Tumor location	0.994	0.990	0.990	0.990
Spiculation	0.979	0.988	0.945	0.966
Vessel invasion	0.982	0.932	0.788	0.854
HLNLA^e^	0.965	1.000	0.811	0.896
HLNSA^f^	0.986	0.982	0.848	0.911

^a^MLNLA: mediastinal lymph node long axis.

^b^TLA: tumor long axis.

^c^TSA: tumor short axis.

^d^MLNSA: mediastinal lymph node short axis.

^e^HLNLA: hilar lymph node long axis.

^f^HLNSA: hilar lymph node short axis.

## Discussion

### Principal Findings

In this study, we explored the feasibility of using EMR to develop machine learning models to predict LNM for patients with NSCLC. The important features about the primary tumor and lymph nodes were extracted from the CT reports using NLP technique to support the model development. To the best of our knowledge, this is the first study to use NLP technique to extract features to build preoperative LNM prediction models for patients with NSCLC. Experimental results indicate that the RF model achieved the best performances with 0.792 AUC value and 0.456 AP value for pN2 LNM prediction. All machine learning models outperformed the size criterion and clinician’s evaluation.

Among all models, the LR, L2-LR, RF, and LGBM provide the feature importance to show the connections between the patient features and LNM status. CEA, tumor density, CA125, MLNLA, TLA, lobulation, and TSA were ranked in the top 10 important features by the machine learning models, which was consistent with the results of univariate analysis. Squamous cell carcinoma antigen (SCCAg) was also identified as a top 10 important feature by the models, although univariate analysis did not show significance. However, SCCAg has been proved to be associated with LNM in esophageal squamous cell carcinoma [46], anus squamous cell carcinoma [47], oral-cavity squamous cell carcinoma [48], and cervical squamous cell carcinoma [49]. It is also a poor prognostic factor of lung squamous cell carcinoma and upgrading the patient stage is recommended [50,51]. Surprisingly, TLA was identified as an important feature with negative weight by the LR model, which means the longer the TLA is, the lower the risk of pN2 LNM the patient may have. The result is contrary to the result of univariate analysis and may be caused by multicollinearity or interactions between the features [52]. In the L2-LR model, the TLA was not ranked in the top 10 important features, indicating the L2 regularization can indeed reduce the influence of multicollinearity and improve the interpretability of the model [53]. In addition, other features like right middle lobe cardiovascular disease also suffered interpretability problems, which may be hard to accept in clinical practice. Therefore, more robust interpretable machine learning algorithms are needed to make accurate predictions while giving more reasonable explanations.

In this study, we innovatively extracted features from CT reports and used them to develop LNM prediction models. The concordance correlations between the predicted probabilities of models using NLP-extracted features, partially NLP-extracted features, and gold standard features indicate that the automatically developed models can obtain similar predictive results to those of models using gold standard features. This finding implicitly indicates it is possible to build models using a large amount of unstructured data and update them automatically. More importantly, it can also reduce the burden of manual feature extraction to improve the usability of the prediction models in clinical practice.

### Limitations

Although the experimental results show that machine learning models using CT reports, demographic information, medical history, and biomarker data can achieve better performances than the size criterion and clinician’s evaluation on the collected data, external validation is still needed to further prove the effectiveness and generalization of the NLP and LNM prediction models. Note that the writing styles of CT reports from different medical centers may vary greatly, which poses a huge challenge to the NLP model developed using the CT reports from a single medical center. Transfer learning is a proper strategy to solve the problem by fine-tuning the model to adapt to CT reports from other centers. Overall, multicenter data is necessary to develop a more robust and generalizable NLP and LNM prediction model.

Furthermore, many studies have proved that there are deep features or radiomics features related to LNM in the CT images [54-60]. Clinicians cannot recognize these with the naked eye, so these features may provide extra information about the metastasis status. In the future, we will extract the image features and combine them with the features in this study to develop more robust, accurate multimodal LNM prediction models.

### Conclusions

In this study, we used NLP and machine learning methods to develop the LNM prediction models for patients with NSCLC using EMRs. The RF model achieved the best performance with 0.792 AUC value and 0.456 AP value for pN2 prediction and 0.768 AUC value and 0.524 AP value for pN1&N2 prediction. All machine learning models outperformed the size criterion and clinician’s evaluation. Furthermore, the experimental results indicate that the NLP model can effectively extract features from CT reports to support the automatic development and update of the LNM prediction model and may facilitate the application of models in clinical practice.

## Abbreviations

ANN: artificial neural network
AP: average precision
AUC: area under the receiver operating characteristic curve
BERT: bidirectional encoder representations from transformers
BiLSTM: bidirectional long short-term memory
BI-RADS: breast imaging-reporting and data system
CA125: carbohydrate antigen 12-5
CEA: carcinoembryonic antigen
cN: clinical N stage
EMR: electronic medical record
LGBM: LightGBM
LNM: lymph node metastasis
LR: logistic regression
L2-LR: L2-logistic regression
MLNLA: mediastinal lymph node long axis
MTQA: multiturn question answering
NLP: natural language processing
NSCLC: non–small cell lung cancer
NSE: neuron-specific enolase
PET-CT: positron emission tomography–computed tomography
pN: pathological N stage
PR: precision-recall curve
RF: random forest
ROC: receiver operating characteristic curve
SCCAg: squamous cell carcinoma antigen
SUVmax: maximum standardized uptake value
SVM: support vector machine
TLA: tumor long axis
TSA: tumor short axis
